# Dietary melanoidins as emerging functional components: interactions with gut microbiota and implications for nutritional modulation of intestinal health

**DOI:** 10.3389/fnut.2025.1672681

**Published:** 2025-12-04

**Authors:** Jialiang Chen, Xiaoyi Gao, Yue Hou, Guowei Liang

**Affiliations:** 1 Department of Clinical Laboratory, Aerospace Center Hospital, Beijing, China; 2 Translational Medicine Center, Beijing Chest Hospital, Capital Medical University, Beijing, China

**Keywords:** melanoidins, gut microbiota, short-chain fatty acids, Maillard reaction, prebiotics

## Abstract

Melanoidins, complex brown polymers formed during the Maillard reaction in thermally processed or fermented foods, are increasingly recognized for their nutritional relevance beyond sensory contributions. Emerging evidence suggests that they may act as prebiotic-like compounds that resist human digestion and undergo microbial fermentation in the colon, producing metabolites such as short-chain fatty acids (SCFAs). These metabolites are proposed to support intestinal barrier function, inflammation, and host metabolism. This review summarizes current knowledge on the gastrointestinal fate, microbial fermentation, and putative bioactivities of dietary melanoidins, with a focus on their interactions with gut microbiota. We compare the structural diversity among food sources and discuss potential health implications. However, most evidence to date derives from *in vitro* and animal studies, with limited clinical validation. Key challenges remain in classification, extraction, and the translation of preclinical findings into human applications. Addressing these gaps will be essential to establish the nutritional potential of melanoidin-rich foods in personalized and preventive nutrition strategies for gut health. Future studies integrating standardized extraction, structural characterization, and clinical validation are essential to establish the role of dietary melanoidins in personalized nutrition.

## 1 Introduction

The human gut harbors a complex and dynamic microbial ecosystem-the gut microbiota-which plays a pivotal role in immunometabolism, nutrient processing, and host defense. Fueled primarily by indigestible dietary components such as non-starch polysaccharides, resistant oligosaccharides, and resistant starch, these microorganisms produce a range of metabolites, including short-chain fatty acids (SCFAs: acetate, propionate, and butyrate) and lipopolysaccharides (LPS), that contribute to maintaining intestinal homeostasis and immune regulation (1). A well-balanced gut microbiota forms a protective ecological barrier, while disruptions in microbial composition-often driven by poor diet-are linked to a range of health disorders. Although the benefits of dietary fibers, polypeptides, saponins, polysaccharides, and probiotics have been widely studied (2), melanoidins have recently emerged as novel modulators of gut health.

Melanoidins are nitrogen-rich brown polymers generated via the Maillard reaction-a non-enzymatic process involving reducing sugars and amino acids under heat. They are widely present in thermally processed or fermented foods such as roasted coffee (3), baked goods (4), grilled meats (5), and fermented foods like soy sauce (6–8). The estimated daily intake of melanoidins is approximately 10–12 g, with only 10–30% being absorbed by the host (9). Once regarded as biologically inert, they are now recognized for their microbiota-mediated health benefits (10).

Accumulating evidence indicates that melanoidins exert antioxidant (11), anti-inflammatory (12), and antimicrobial properties (13). *In vitro* and animal studies report shifts in microbial taxa (e.g., increases in Bifidobacterium) after exposure to melanoidin-rich substrates (14). Upon reaching the colon undigested, melanoidins are fermented by gut microbes, producing SCFAs such as acetate, propionate, and butyrate (15). These metabolites contribute to improved mucosal barrier function, reduced inflammation, and provide energy for colonocytes. Melanoidin-rich foods such as coffee, whole grains, and roasted vegetables may therefore support a healthier gut microbiome and overall immune function (16).

Despite promising findings, the structural diversity of melanoidins-affected by food source and processing conditions-and individual differences in microbiota composition may lead to variable biological outcomes. Understanding these interactions is essential for unlocking their full therapeutic potential. In this review, we explore the formation, gastrointestinal fate, microbial fermentation, and health-related effects of dietary melanoidins. We aim to provide a comprehensive overview that supports future applications in functional food design and the dietary management of gut-related diseases such as inflammatory bowel disease (IBD), irritable bowel syndrome (IBS), and colorectal cancer (13).

## 2 Methods

The manuscript is presented as a narrative review with transparent literature-search and selection procedures. We searched PubMed, Scopus, Web of Science, and Google Scholar for articles published up to 31 May 2025. Search terms combined keywords and synonyms for melanoidins and gut microbiota, such as “melanoidins,” “Maillard reaction products,” “gut microbiota,” “melanoidin fermentation,” “SCFAs,” “functional foods,” “intestinal health,” and “melanoidin metabolism.” No language restriction was applied at the search stage; however, only articles with an English abstract were screened. Titles and abstracts were independently screened by two reviewers (J. C. and X. G.), and full texts were retrieved for potentially relevant citations. Inclusion criteria were: (i) original experimental studies (*in vitro*, animal *in vivo*, human intervention/observational) examining melanoidins or foods explicitly characterized as melanoidin-containing; (ii) mechanistic studies addressing melanoidin digestion, microbial metabolism, or physiological effects plausibly linked to melanoidins; (iii) reviews addressing melanoidins when providing mechanistic or compositional synthesis. Because the work is a narrative review synthesizing heterogeneous evidence across model systems, no meta-analysis was attempted.

## 3 The formation and composition of melanoidins

Melanoidins are complex, bioactive compounds formed through the Maillard reaction between reducing sugars and amino acids during food processing (17). They contribute not only to the characteristic color, flavor, and aroma of cooked foods, but are also increasingly associated with several health benefits (17).

**Figure 1:**
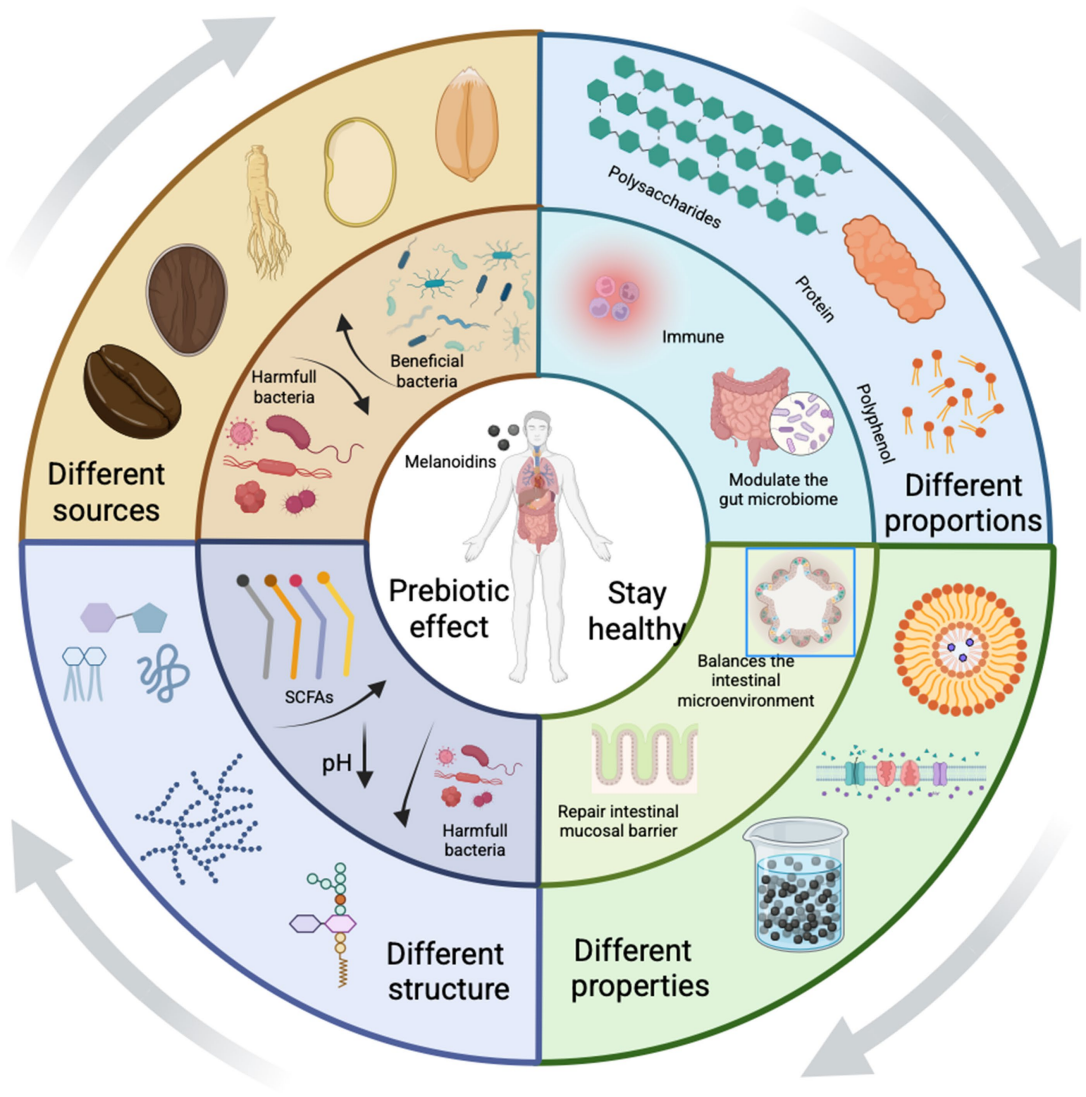
Melanoidins influence gut homeostasis. Melanoidins promote gut health via four main pathways: (a) modulation of microbial composition (increasing Bifidobacterium and Lactobacillus while reducing pathogenic taxa), (b) reinforcement of intestinal barrier function through mucin and tight junction proteins, (c) regulation of intestinal pH that favors beneficial microbes, and (d) exertion of anti-inflammatory and antioxidant effects. Created using Biorender, licensed under Academic License.

Growing evidence indicates that melanoidins are not inert but may actively promote gut homeostasis (15), which encompasses a balanced microbiota, intestinal barrier integrity, immune regulation, and metabolic health. As summarized in Figure 1, melanoidins support gut homeostasis through multiple mechanisms: (a) Modulating gut microbial composition: They can alter the balance of beneficial and harmful bacteria in the gut, fostering a healthier microbiome. (b) Regulating intestinal homeostasis: Melanoidins have anti-inflammatory properties that help mitigate chronic gut inflammation. (c) Supporting the intestinal mucosal barrier: They enhance the strength and function of the intestinal lining, preventing the leakage of harmful substances into the bloodstream. (d) Adjust intestinal pH value: They help maintain an optimal pH in the intestines, creating an environment that favors beneficial bacteria and inhibits pathogenic organisms.

The health effects of melanoidins vary by dietary source due to structural differences and food matrix influences (Table 1). While a food-source categorization (e.g., soy, grain) offers an intuitive framework, it may obscure the fundamental structural heterogeneity of melanoidins. Their functional properties are ultimately governed by structural features such as molecular weight, the nature of incorporated phenolic compounds, and the polysaccharide-to-protein ratio, which vary not only between sources but also within processing conditions of the same food. Therefore, the following discussion, while organized by common dietary sources, will emphasize these underlying structural and functional characteristics.

**Table 1:** The chemical composition of melanoidins.

Source	Protein (%)	Polyphenol (%)	Polysaccharides (%)	Melanoidins	Reference
Soy sauce (1–3 kDa)	5.02 ± 0.31	0.20 ± 0.05	1.60 ± 0.06	0.31 ± 0.07 g/100 mL	(93)
Soy sauce (3–10 kDa)	5.08 ± 0.27	0.56 ± 0.10	1.71 ± 0.13	0.42 ± 0.09 g/100 mL	(93)
Soy sauce (10–30 kDa)	7.18 ± 0.40	0.88 ± 0.12	1.73 ± 0.11	0.20 ± 0.05 g/100 mL	(93)
Soy sauce (30–50 kDa)	8.32 ± 0.45	0.55 ± 0.08	2.22 ± 0.16	0.61 ± 0.10 g/100 mL	(93)
Soy sauce (>50 kDa)	10.52 ± 0.72	0.43 ± 0.06	2.83 ± 0.19	0.90 ± 0.13 g/100 mL	(93)
Soy sauce	18.36 ± 0.57	1.45 ± 0.15	5.32 ± 0.24	2.44 g/100 mL	(93)
Dark beer	5.02 ± 0.03	2.91 ± 0.36	71.78 ± 4.46	1.23 ± 0.06 g/100 mL	(18)
Dark beer (APE-M)	2.57 ± 0.00	1.41 ± 0.02	7.89 ± 0.48	12.33 ± 0.60%	(19)
Dark beer (MAE-M)	6.40 ± 0.10	1.76 ± 0.02	NA	13.63 ± 0.34%	(19)
Vinegar	1.20 ± 0.04	4.64 ± 0.52	51.01 ± 0.54	9.30 ± 0.27%	(5)
Spent grain (RF1)	1.42 ± 0.06	0.52 ± 0.01	13.19 ± 0.46	0.97 ± 0.19%	(20)
Spent grain (RF2)	5.96 ± 0.24	0.87 ± 0.01	6.18 ± 0.20	7.24 ± 0.37%	(20)
Spent grain (RF3)	5.00 ± 0.45	0.56 ± 0.01	3.30 ± 0.10	1.22 ± 0.16%	(20)
Spent grain (RF4)	8.89 ± 0.39	1.83 ± 0.12	16.43 ± 0.33	17.43 ± 0.58%	(20)
Chinese liquor (MAF)	30.86 ± 0.52	4.07 ± 0.31	44.73 ± 1.08	17.03 ± 0.41%	(94)
Chinese liquor (MBF)	23.57 ± 0.24	2.76 ± 0.16	56.02 ± 0.50	13.36 ± 0.55%	(94)
Chinese liquor (MAD)	32.78 ± 0.64	4.27 ± 0.26	56.53 ± 1.22	26.86 ± 0.74%	(94)
Black wolfberry (<3 kDa)	1.05 ± 0.07	0.25 ± 0.01	6.09 ± 0.60	24.21 ± 3.01%	(95)
Black wolfberry (3–10 kDa)	1.10 ± 0.03	0.26 ± 0.01	7.14 ± 0.31	59.37 ± 3.66%	(95)
Black wolfberry (10–50 kDa)	1.15 ± 0.03	0.30 ± 0.01	5.91 ± 0.24	12.90 ± 1.25%	(95)
Black wolfberry (>50 kDa)	1.26 ± 0.03	0.49 ± 0.01	7.06 ± 0.14	3.52 ± 0.98%	(95)
Black ginseng	0.87 ± 0.03	NA	13.12 ± 0.39	72.54 ± 0.62%	(88)

Units in this table have been standardized to percentage (%) for solid samples or g/100 mL for liquids to enable cross-comparison. Original reported units are provided in Supplementary Table S1. “NA” indicates data not available in the cited source. Please refer to individual references for detailed extraction and analytical methods. MAE, macroporous resin adsorption extraction; APE, acetone precipitation extraction; RF1, aqueous-extracted fraction; RF2, 1 M NaOH-extracted fraction after 110 mM NaOH extraction; RF3, aqueous-washed fraction after 1 M NaOH extraction; RF4, 110 mM NaOH-extracted and isoelectrically precipitated fraction; MBF, melanoidins before fermentation; MAF, melanoidins after fermentation (before distillation); MAD, melanoidins after distillation.

As summarized in Table 1, the composition of melanoidins varies dramatically across sources. For instance, soy sauce melanoidins are characterized by a relatively high protein content (>5%), whereas melanoidins from dark beer and vinegar are exceptionally rich in polysaccharides (>50%), a feature that likely dictates their strong prebiotic potential (18, 19). The notable variation in polyphenol incorporation, as seen in vinegar (4.64%) compared to spent grain fractions (0.52–1.83%), may contribute to differences in their antioxidant capacities (5, 20). The significant impact of extraction protocols and the food matrix on these compositional profiles underscores that source alone is an inadequate predictor of function. Thus, moving beyond a simplistic food-category classification to a structure- and function-centric understanding is crucial for harnessing the full potential of melanoidins in gut health.

**Figure 2:**
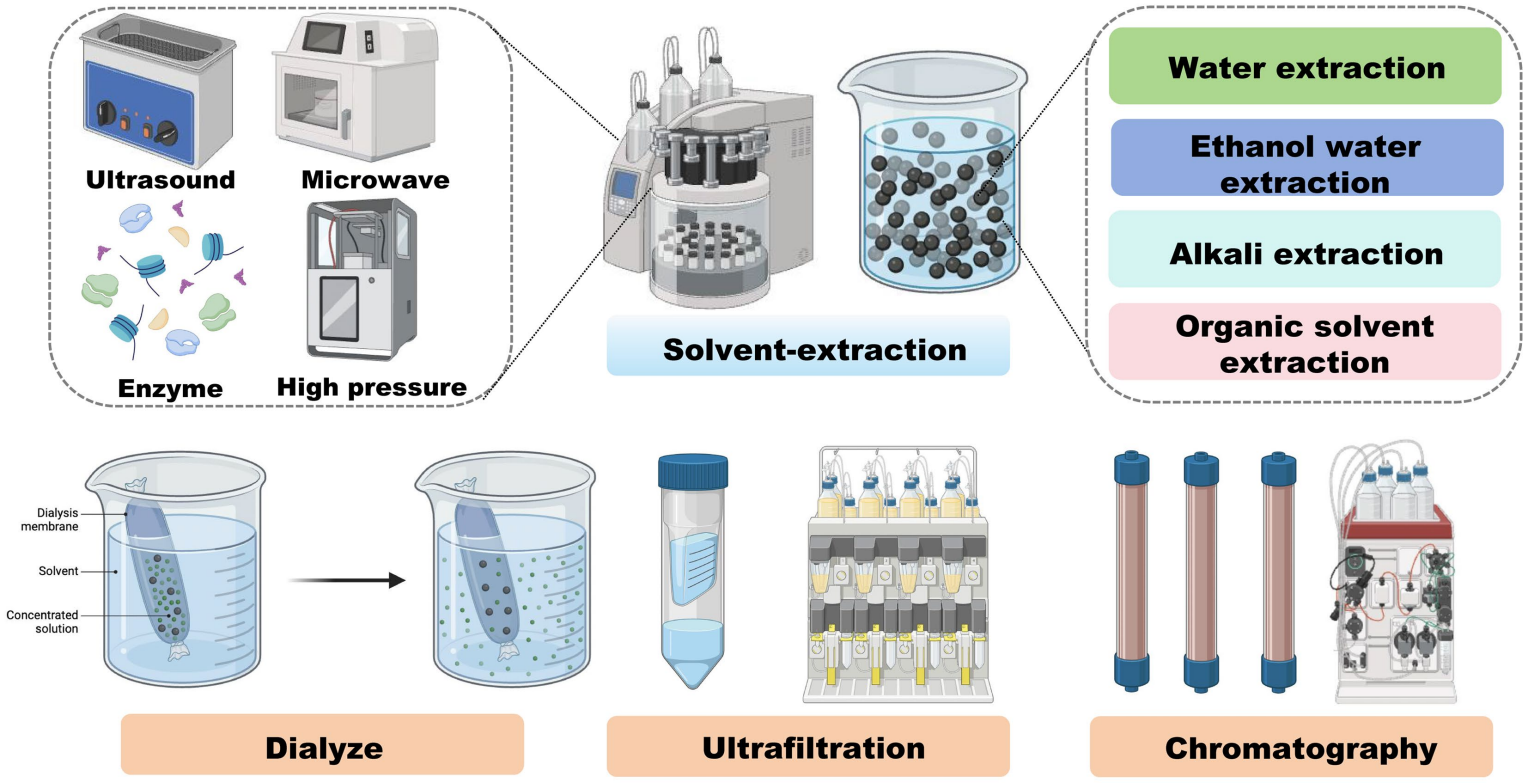
The formation and extraction of melanoidins. Workflow summarizing the Maillard reaction during food processing (soy, grains, coffee, vinegar, etc.) leading to melanoidin formation, followed by extraction methods (aqueous/alkaline extraction, ultrafiltration, chromatography) and structural characterization (HPLC, MS). Created using Biorender, licensed under Academic License.

The main processes and analytical techniques applied to isolate and characterize melanoidins are summarized in Figure 2, which highlights the methodological variability contributing to heterogeneous study outcomes. Typical protocols involve aqueous or alkaline solvent extraction under heating, followed by centrifugation and filtration. Further purification may involve dialysis, ultrafiltration, or chromatography (e.g., size-exclusion or ion-exchange). Isolated melanoidins are then characterized using advanced tools such as HPLC and mass spectrometry.

Efficient extraction and separation are essential to advance our understanding of melanoidin structures and their specific interactions within the gut environment, ultimately elucidating their role in sustaining gut homeostasis.

### 3.1 Melanoidins from plant-based sources: soy and legumes

Soybeans are a dietary staple rich in bioactive compounds, including reducing sugars (21), proteins, and isoflavones (2). Soy-based melanoidins are formed when these components undergo the Maillard reaction during heating, cooking, or fermentation (22). Unlike some other melanoidins, they are derived from a plant-based matrix that also contains fiber, polyphenols, and antioxidants, enhancing their potential bioactivity.

These melanoidins, abundant in fermented soybean products such as soy sauce, douchi, and miso, exhibit several gut health-promoting properties. They function as prebiotics by selectively stimulating beneficial bacteria like Bifidobacterium and Lactobacillus, thereby supporting microbial balance and reducing dysbiosis risk (23). Additionally, soy melanoidins demonstrate anti-inflammatory activities that may alleviate gut inflammation related to IBS or IBD (24).

Their antioxidant properties further contribute to gut protection by mitigating oxidative stress, which can compromise intestinal barrier integrity and lead to conditions such as leaky gut syndrome (25). Through these combined mechanisms, soy-based melanoidins help maintain mucosal defense and overall gut homeostasis.

### 3.2 High fiber associated melanoidins: cereals and grains

Grain-based foods such as whole wheat, barley, oats, and rice are excellent sources of dietary fiber, vitamins, and minerals (26). During their processing and cooking, the Maillard reaction occurs, leading to the formation of melanoidins. These melanoidins, found in roasted, baked, or fermented grains, possess a distinctive chemical structure that can specifically influence gut homeostasis. Typically, polysaccharide-based, grain-derived melanoidins are commonly present in products like dark beer (19), vinegar (27), and black bread (28).

Grain-based melanoidins are generally more resistant to digestion by human enzymes, meaning they are likely to reach the colon, where they interact with the gut microbiota (29). Studies have shown that grain-derived melanoidins can act as prebiotics, promoting the growth of beneficial gut bacteria, particularly fiber-fermenting bacteria that contribute to the production of SCFAs (30). Additionally, grain-based melanoidins may help regulate the microbiota’s composition by enhancing the growth of beneficial microbes while inhibiting the growth of harmful bacteria (31). This regulation helps maintain microbial diversity, which is essential for the optimal functioning of the gut microbiome. Grain-based melanoidins, therefore, contribute to gut homeostasis by fostering a balanced microbial environment and supporting the production of metabolites that promote intestinal health. Furthermore, grain-based melanoidins have been shown to possess antioxidant (32) and anti-inflammatory properties (33), which may help mitigate oxidative stress and inflammation within the gut. This action could be particularly beneficial in protecting against chronic diseases, such as colorectal cancer or metabolic disorders, that are associated with gut inflammation and dysbiosis.

### 3.3 Diverse source melanoidins: coffee, fruits, and herbs with distinct bioactivities

Melanoidins are also derived from diverse sources beyond soy and grains, including coffee, roasted fruits, processed herbs, and fermented blackened foods (6). Their structural and functional properties vary considerably depending on raw materials and processing conditions, leading to distinct impacts on gut health (34). For instance, coffee melanoidins are formed during the roasting process, which involves high temperatures that promote extensive Maillard reactions, resulting in complex polymers rich in aromatic compounds (35). Similarly, roasted fruits such as apples (36) and potatoes (37) develop melanoidins that retain some of their original nutrients while acquiring new bioactive properties. Processed herbs, such as ginseng (38), wolfberry (39), ophiopogonis (40), rehmannia glutinosa (41), etc., contain melanoidins that contribute to their characteristic dark color and robust flavors, while also offering potential prebiotic effects that support beneficial gut bacteria. The chemical structure of melanoidins varies significantly across these different sources, leading to diverse biological activities. For example, coffee melanoidins are known for their high antioxidant capacity (32), which can help reduce oxidative stress in the gut. In contrast, melanoidins from roasted cocoa may be more effective in modulating specific microbial populations (42). These functional differences underscore the need for source-specific research to elucidate how different melanoidins interact with the gut environment. Investigating a broader range of melanoidin-rich materials will facilitate their targeted use in promoting microbiome balance and gut health.

In conclusion, the diverse sources and structural complexity of melanoidins underlie their multifaceted roles in modulating gut homeostasis. The melanoidins exert distinct prebiotic, anti-inflammatory, and antioxidant effects, largely mediated through microbial fermentation and metabolic activity in the colon (43). These interactions enhance beneficial microbiota, strengthen intestinal barrier function, and mitigate inflammation-key mechanisms in maintaining gut health (44). However, the efficacy and physiological impact of melanoidins are influenced by their food matrix, chemical structure, and individual gut microbiota composition. However, the effects of different sources vary significantly, but the research methods are inconsistent, making it difficult to compare the results horizontally. Future research should prioritize human trials and multi-omics approaches to clarify dose–response relationships, individual variability, and the synergistic effects of melanoidins with other dietary components. Such insights will be essential for developing targeted nutritional strategies to prevent gut-related disorders and promote health through diet.

## 4 The biological fate of dietary melanoidins through the gastrointestinal tract

Dietary melanoidins, complex nitrogen-rich compounds formed during the Maillard reaction in foods like roasted coffee, baked goods, and grilled meats, play an intriguing role in human health (45). Unlike many other dietary components, melanoidins are not fully digested by human enzymes in the gastrointestinal tract (46). Instead, they interact with the gut microbiota, which plays a key role in their breakdown and metabolism (47). The biological fate of dietary melanoidins involves several stages in the gastrointestinal tract, where they undergo digestion, fermentation, and modification by microbial activity (48). These processes lead to the formation of various metabolites that can influence gut health, immune function, and even systemic metabolism. Current evidence on the gastrointestinal processing of dietary melanoidins comes predominantly from *in vitro* simulated digestion and fermentation studies, complemented by animal experiments; human *in vivo* digestion data are scarce.

### 4.1 Digestion of melanoidins in the stomach and small intestine

**Figure 3:**
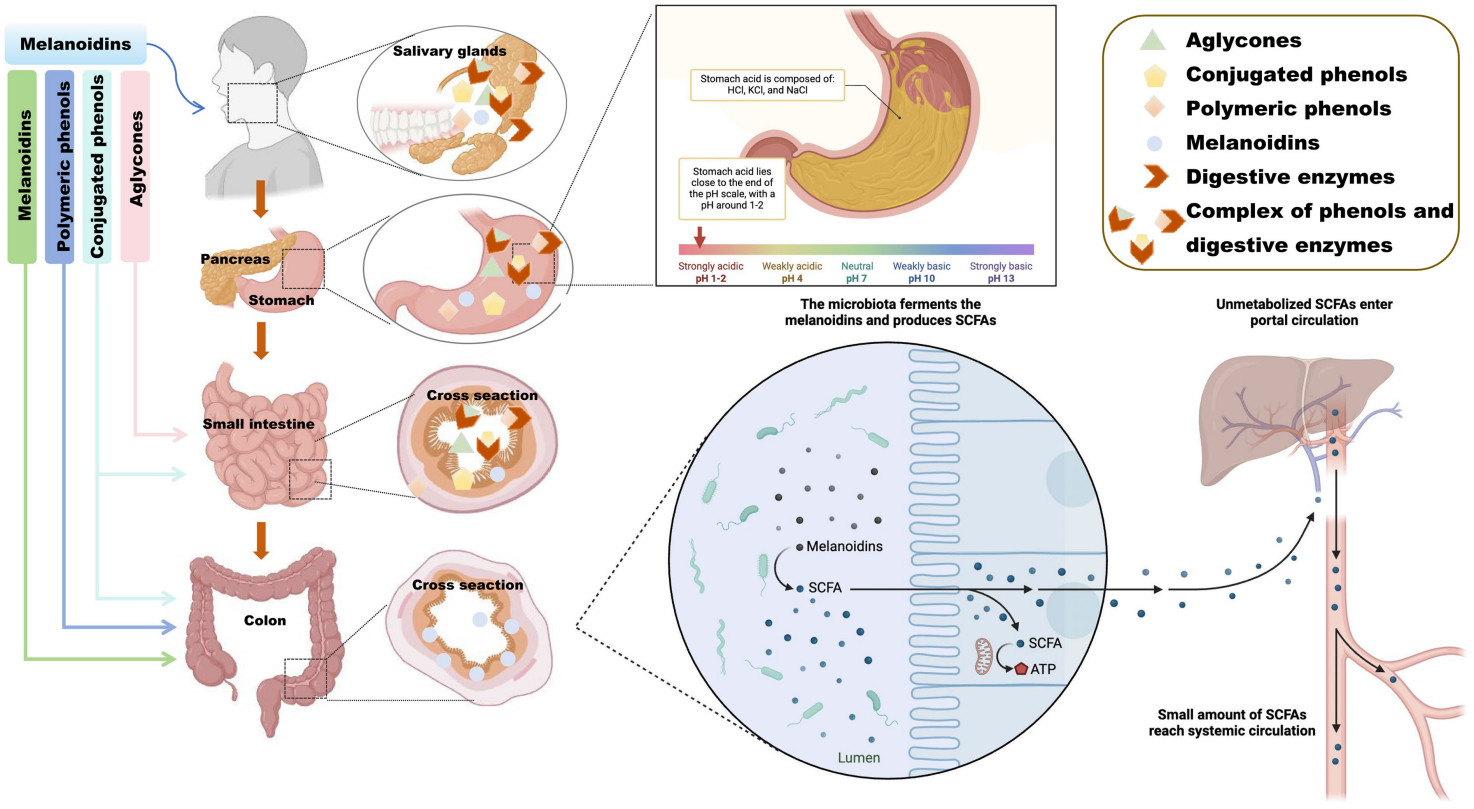
Digestive mechanism diagram of melanoidins. Schematic illustration showing the resistance of melanoidins to enzymatic digestion in the stomach and small intestine, followed by their arrival in the colon where gut microbiota metabolizes them into bioactive compounds, including short-chain fatty acids (SCFAs) and phenolic derivatives. The figure highlights two key roles: (1) melanoidins as carriers for polyphenols, protecting them from early degradation, and (2) their fermentation by colonic microbes, leading to SCFA production that supports intestinal health. Created using Biorender, licensed under Academic License.

Upon ingestion, melanoidins enter the stomach and are exposed to its acidic environment (pH 1.5–3.5), which facilitates the release of bound phenolics that may exert bioactivity locally (49). Nevertheless, melanoidins remain largely resistant to human digestive enzymes such as amylases, lipases, and proteases (28). As shown in Figure 3, they pass undigested through the stomach and small intestine, where pancreatic enzymes also fail to hydrolyze them significantly (50, 51).

Thus, most melanoidins proceed intact to the colon and become available for microbial fermentation. The gut microbiota possesses specialized enzymes that degrade melanoidins into absorbable metabolites with potential bioactivity (52). For example, roasted coffee-rich in melanoidins-shows stronger antioxidant activity than green coffee, partly due to Maillard reaction products and modified phenolic profiles (53). Although fermented green coffee yields more total SCFAs, melanoidins in roasted coffee may enhance bioavailability and microbial metabolism through two key mechanisms: acting as carriers that facilitate polyphenol release and absorption in the small intestine, and serving as fermentable carbon sources for colonic microbiota, thereby promoting SCFA production (33).

### 4.2 Melanoidins metabolism by gut microbiome

The gut microbiota plays a central role in the metabolism of melanoidins (54). Although these compounds are resistant to human digestive enzymes, many gut bacteria can ferment or modify them. Melanoidins can be utilized by gut microbes, leading to the production of SCFAs and shaping their community structure. Many melanoidins promote the growth of beneficial genera such as *Bifidobacterium* and *Faecalibacterium* (30). By fermenting melanoidins, gut microbes can release some phenolic compounds initially linked to the melanoidin backbone, which in turn can enhance the absorption of phenolics. Analysis of these polyphenols can be used to investigate the structure of melanoidins and to explore microbial metabolic pathways (17). The breakdown of melanoidins by gut microbes results in the production of various metabolites that significantly impact intestinal health and overall physiological functions (55). While the direct decomposition of melanoidins primarily releases phenolic metabolites, it also significantly stimulates the growth of SCFA-producing bacteria, leading to an increased production of short-chain fatty acids (SCFAs) (6). These substances have a systemic impact on the host’s energy balance, inflammatory response, and lipid metabolism by regulating the structure and metabolic function of the intestinal flora.

#### 4.2.1 Melanoidin-specific metabolites

##### 4.2.1.1 Phenolic compounds

Polyphenols represent important structural components within melanoidins. Although melanoidins are generally resistant to digestion, they can release polyphenols through microbial fermentation in the colon, allowing these bioactive compounds to exert systemic effects (52). Melanoidins thereby function as a natural carrier for polyphenols, improving their stability and bioavailability throughout the gastrointestinal tract (56). This binding protects polyphenols from premature degradation by digestive enzymes and enables their targeted release in specific gut regions. The gradual liberation of polyphenols from melanoidins supports sustained antioxidant and anti-inflammatory activities, which are crucial for maintaining gut health. Furthermore, melanoidin-bound polyphenols play a key role in modulating the gut microbiota. They selectively stimulate beneficial bacteria, including Firmicutes and Bifidobacterium, while suppressing pathogenic species, thereby promoting microbial balance and helping to prevent dysbiosis-related disorders (57). Additionally, the antioxidant properties of polyphenols derived from melanoidins help mitigate oxidative stress within the intestinal environment, protecting epithelial cells from damage and supporting the maintenance of the intestinal barrier function (43). By reducing oxidative stress and inflammation, these compounds play a crucial role in preventing the onset and progression of IBD and IBS.

Future research should focus on elucidating the specific mechanisms through which melanoidin-bound polyphenols interact with the gut microbiota and host cells. Understanding these interactions will provide deeper insights into the development of functional foods and therapeutic strategies aimed at enhancing gut health and overall well-being.

#### 4.2.2 General microbial metabolites potentially influenced by melanoidins

##### 4.2.2.1 SCFAs

The microbial fermentation of melanoidins yields SCFAs-mainly butyrate, acetate, and propionate-which play essential roles in gut health (2, 58). Butyrate serves as the primary energy source for colonocytes, strengthens the intestinal barrier, and exhibits anti-inflammatory effects (59). Acetate and propionate contribute to lipid metabolism and modulate gut microbial composition (47). By lowering colonic pH, SCFAs also inhibit pathogens while supporting beneficial bacteria (34).

As signaling molecules, SCFAs bind to receptors such as GPCR41 and GPCR43, helping to regulate immune and metabolic pathways—including cytokine production and inflammation control—which may protect against chronic diseases (60–62). SCFAs also influence the gut–brain axis; butyrate, for example, helps maintain blood–brain barrier function and reduce neuroinflammation, thereby potentially supporting cognitive health (63, 64). Furthermore, SCFAs contribute to appetite regulation and energy homeostasis, suggesting relevance in managing obesity (65).

The benefits of melanoidin fermentation, however, vary with diet, microbial diversity, and other bioactive compounds (29). Future studies should clarify the mechanisms behind melanoidin-host interactions and explore personalized nutrition strategies to maximize their health potential (66).

##### 4.2.2.2 Ammonia

Ammonia is another byproduct of the microbial fermentation of proteins and complex compounds like melanoidins (51). Some bacteria in the gut, particularly proteolytic bacteria, break down amino acids and peptides, releasing ammonia as a byproduct. While small amounts of ammonia are used by the body for the synthesis of urea, excessive ammonia can be toxic and is implicated in gut dysfunction, such as in IBD (67). However, the production of ammonia from melanoidins may be influenced by the specific microbial communities present in the gut, with some strains of bacteria being more efficient at producing ammonia than others (68). In addition, ammonia can also serve as one of the precursors for the formation of melanoidins. The processes of its formation and release may undergo dynamic changes within the body (69), thereby affecting the physicochemical properties of melanoidins (70).

##### 4.2.2.3 BAs (biogenic amines)

BAs, such as putrescine, cadaverine, and histamine, are another class of metabolites produced by the gut microbiota during the fermentation of dietary compounds like melanoidins (71–73). These amines are formed from the decarboxylation of amino acids and are associated with both beneficial and detrimental effects on health (74). At low concentrations, BAs can have positive effects on gut health, such as acting as signaling molecules that regulate gut motility and microbial interactions (75). However, excessive levels of biogenic amines, particularly histamine, can lead to adverse effects such as headaches, digestive disturbances, and allergic reactions (76). Melanoidins are enriched in soy-based fermented foods, and the control of BAs is crucial to ensure the safety of fermented soybean products (22).

##### 4.2.2.4 ICs (indolic compounds)

ICs are produced from the microbial fermentation of tryptophan, an essential amino acid that is present in melanoidins (77, 78). These compounds, including indole and its derivatives, can influence gut health by acting on the gut-brain axis (79). Some ICs have been shown to have neuroactive properties, affecting mood and cognitive function (80). In the gut, indolic compounds can also modulate inflammation and microbial activity, playing a role in maintaining gut homeostasis. However, imbalances in the production of indolic compounds may contribute to the pathogenesis of gastrointestinal disorders.

##### 4.2.2.5 H_2_S

H_2_S is a gas that is produced by certain gut bacteria during the fermentation of sulfur-containing compounds, including those found in melanoidins (81). While H_2_S is often considered a toxic compound due to its strong odor and association with gut dysfunction, recent studies have shown that it also has beneficial roles in gut health (82). At low concentrations, H_2_S acts as a signaling molecule, helping to regulate mucosal integrity, promote blood flow to the colon, and modulate inflammation (83). However, excessive production of H_2_S can lead to adverse effects, such as damage to intestinal cells and dysbiosis (microbial imbalance).

##### 4.2.2.6 Other GMMs

In addition to the metabolites mentioned above, the fermentation of melanoidins can lead to the production of a wide range of other GMMs that may influence health (84). These include various organic acids, gases (e.g., methane and carbon dioxide), and peptide derivatives that can impact gut pH, microbial composition, and overall gut function (26). The complex interplay between these metabolites can influence the gut’s immune response, nutrient absorption, and protection against pathogenic bacteria.

In summary, phenolic compounds are directly released from the melanoidin structure and are thus considered core melanoidin-specific metabolites. In contrast, other metabolites such as SCFAs, ammonia, BAs, ICs, and H_2_S represent general microbial fermentation products. Their production can be influenced by melanoidins, but they are not unique to melanoidin metabolism, as they are derived from common pathways involving proteins, amino acids, and carbohydrates (85). Although existing studies have indicated that melanoidins can release various metabolites through microbial fermentation in the colon, and potentially influence gut health via modulation of the gut microbiota, immune responses, and metabolic pathways, significant knowledge gaps remain regarding their precise mechanisms of action and overall physiological effects (86). Current evidence is largely derived from *in vitro* or animal models, with a lack of validation in humans, particularly across individuals with varying health statuses. Future research should integrate multi-omics technologies, *in vitro* fermentation models, and human intervention trials to systematically elucidate the relationship between melanoidins and host-microbiota interactions, with emphasis on their potential application in personalized nutrition and functional food development.

## 5 Effect of melanoidins on gut-associated diseases

**Figure 4:**
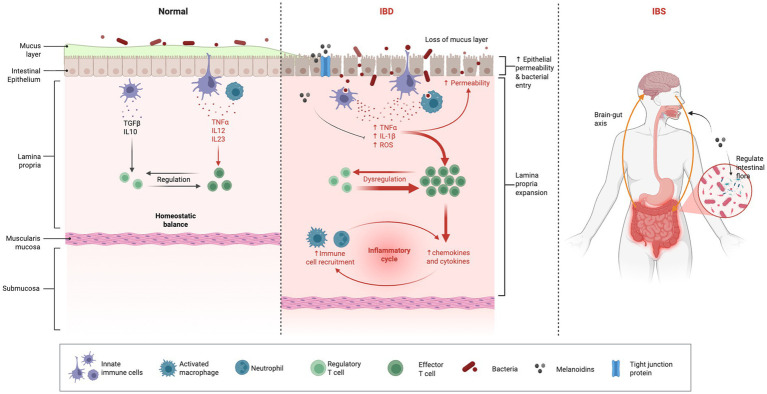
Effect of melanoidins on gut homeostasis. Diagram showing how melanoidins may alleviate IBD and IBS by restoring microbial balance, enhancing SCFA production, reducing oxidative stress, and reinforcing mucosal barrier function. Created using Biorender, licensed under Academic License.

The gut is a complex and dynamic environment where microbial, immune, and epithelial cells interact to maintain health. However, disturbances in this balance can lead to gut-associated diseases (Table 2), such as IBD and IBS. Melanoidins, complex compounds formed during the Maillard reaction in various foods, have emerged as potential modulators of gut health (30). Their influence on the gut microbiota, intestinal barrier integrity, and immune responses suggests that they could play a beneficial role in the prevention and management of these gut-associated diseases. This section explores the potential effects of melanoidins on two common gastrointestinal conditions: IBD and IBS (Figure 4).

**Table 2:** Potential biological and functional properties of melanoidins.

Source	Function property	Model	Mechanism	Reference
Human (interventional)				
Bread	Anti-obesity	Healthy subjects	Lowering blood toxins, liver fat, and the development of fatty liver	(4)
Coffee	Anti-obesity	Healthy subjects	Lowering energy intake	(4)
Animal (mouse)				
Barley	Antioxidant	*Digestive model*	Promote the growth of Lactobacilli and Bifidobacteria in the gastrointestinal tract, preventing the colonization of potential pathogens.	(30)
Vinegar	Immunomodulatory effects	Cyclophosphamide-induced immunocompromised mouse model	Adjusted immune factors and the levels of Muc-2, Occludin, and Claudin-1, and restored the balance of gut bacteria to support immune function.	(96)
Vinegar	Prebiotic	Alcohol-induced liver damage	Balances gut bacteria and maintains a healthy intestine.	(92)
Soybean	Prebiotic	D-galactose-induced ICR mice	Enhances the balance of gut bacteria and their byproducts while reducing oxidative stress, memory problems, and overall inflammation.	(23)
Black garlic	Anti-obesity	High-fat diet-induced obese C57BL/6 J mice	Reduces short-term hunger and increases feelings of fullness.	(6)
Black garlic	Prebiotic	High fat diet induced dysrhythmia of intestinal microorganisms	Within one day, SCFAs-producing bacteria increase, and inflammation-related bacteria become more stable.	(97)
Soybean	Antioxidant	Aging ICR mice	Food intake, body weight, and organ sizes returned to normal. Feces became darker and urine brighter. Liver MDA levels decreased, and blood antioxidants increased. The ratio of Bacteroidetes to Firmicutes bacteria went down, Lactobacillus levels rose, and harmful bacteria like Porphyromonadaceae decreased.	(98)
*In vitro*				
Miso	Anticancer activity	Human colon carcinoma cell and human gastric carcinoma cell	Growth inhibition effect	(99)
Soybean sauce	Anticancer activity	Human cloned colon adenocarcinoma cells	Growth inhibition effect	(100)
Grain	Prebiotic	Digestive model *in vitro*	Gut bacteria ferment fiber-like substances in the colon and produce SCFAs.	(17)
Vinegar	Prebiotic	Digestive model *in vitro*	Supports gut health by increasing beneficial bacteria like Firmicutes and Bifidobacterium.	(57)
Cocoa	Prebiotic	Digestive model *in vitro*	Produce more short-chain fatty acids	(42)

### 5.1 IBD

IBD, including Crohn’s disease and ulcerative colitis, involves chronic inflammation of the gastrointestinal tract driven by dysregulated immune responses, genetic susceptibility, environmental triggers, and gut dysbiosis (Figure 2). Melanoidins offer potential therapeutic benefits in IBD due to their antioxidant (45), anti-inflammatory (27), and prebiotic properties, although current evidence is primarily derived from preclinical studies.

Studies using *in vitro* and animal models indicate that melanoidins can alleviate gut inflammation and oxidative stress (87). They have been shown to inhibit pro-inflammatory cytokines (e.g., TNF-*α*, IL-1β) and suppress key signaling pathways such as NF-κB (Nuclear Factor Kappa-Light-Chain-Enhancer of Activated B Cells) and AMPK (Adenosine 5’-Monophosphate-Activated Protein Kinase) (27, 88, 89). Additionally, melanoidins act as prebiotics, promoting beneficial bacteria like Bifidobacterium and Lactobacillus, which produce short-chain fatty acids such as butyrate that are known to enhance barrier function and dampen inflammation (17, 90), potentially enhance intestinal barrier integrity, reduce inflammation, and support mucosal immunity. By restoring microbial balance, melanoidins could help address dysbiosis, a central feature of IBD.

Melanoidins also show potential in strengthening intestinal barrier integrity by stimulating mucin secretion and tight junction proteins (e.g., ZO-1, occludin, claudin-1) in preclinical models. This may reduce permeability and prevent the translocation of pathogens and toxins (91). For instance, vinegar melanoidins were shown to improve gut microbiota composition, inhibit ROS, and suppress pro-inflammatory factors in alcohol-treated mice (92).

However, it is crucial to interpret these findings with caution. The majority of evidence comes from animal models, which cannot fully recapitulate the complexity of human IBD. Extrapolating these results directly to human patients is premature. Overall, while more clinical studies are needed to fully understand the therapeutic potential of melanoidins in IBD, their antioxidant, anti-inflammatory, and microbiota-modulating effects suggest that they may be a promising dietary intervention for alleviating the symptoms and reducing the risk of flare-ups in IBD patients.

### 5.2 IBS

IBS is a functional gastrointestinal disorder characterized by chronic abdominal pain, bloating, and altered bowel habits-such as diarrhea, constipation, or mixed patterns-without overt inflammation or structural damage. Its pathophysiology involves disrupted gut motility, visceral hypersensitivity, and gut-brain axis dysfunction, often triggered by stress, diet, or gut microbiota imbalance.

Melanoidins may potentially alleviate IBS symptoms through multiple pathways. For instance, black garlic melanoidins have been shown to modulate gut microbiota and reduce systemic inflammation in obese mice (6). By promoting SCFA-producing bacteria, melanoidins could theoretically support normal gut motility and reduce visceral hypersensitivity, thereby improving bloating and abdominal pain. A study in humans indicated that melanoidin intake influences postprandial appetite-regulating peptides and gut-brain signaling molecules (4), which might indirectly relate to gut function and sensation; however, this study was not specifically designed in IBS patients and its direct relevance to core IBS symptoms remains to be established.

However, clinical evidence remains limited, with most data derived from animal or *in vitro* studies. Individual variations in microbiota and diet also limit generalizability. Moreover, excessive consumption of melanoidins-especially from certain sources-might stimulate harmful microbial metabolites, highlighting the need for further dose–response and safety studies.

## 6 Conclusions and perspectives

Melanoidins, widely present in heat-processed and fermented foods, are emerging as promising functional food components with diverse biological activities observed primarily *in vitro* and in animal models. Their resistance to host digestion and subsequent fermentation by gut microbiota suggests a potential mechanism to influence microbial composition and metabolism, particularly through the generation of SCFAs. Numerous preclinical studies have demonstrated that melanoidins can exhibit antioxidant, anti-inflammatory, and prebiotic properties, which may support gut barrier integrity and microbial homeostasis. These preliminary attributes suggest a potential role in the dietary management of gut-associated disorders such as IBD and IBS that warrants further investigation. However, it is premature to overstate their clinical potential, as the current understanding of melanoidin-microbiota interactions remain constrained by significant methodological challenges. Foremost among these is the lack of standardized methods for melanoidin extraction and characterization from complex food matrices, which leads to poorly defined test materials and hinders cross-study comparisons. Furthermore, the substantial variability in melanoidin structure-dependent on food origin and processing conditions-coupled with inter-individual differences in microbiota composition and complex metabolic pathways, poses considerable challenges for predicting consistent health outcomes in humans.

Therefore, future research must first prioritize overcoming these fundamental hurdles. The immediate path forward should focus on the development of standardized, reproducible protocols for the extraction and characterization of food-derived melanoidins to establish a reliable foundation for subsequent research. Building upon this, well-controlled dose–response studies in animal models are essential to establish causal relationships and define effective doses. Ultimately, these efforts must be translated into rigorous human trials, particularly randomized controlled trials, to validate the prebiotic and anti-inflammatory effects observed preclinically and to assess long-term safety and efficacy. Concurrently, investigating the synergistic effects of melanoidins within a whole-diet context, including their interactions with other dietary components like polyphenols and probiotics, will be crucial. Integrating these mechanistic and clinical insights will be essential to translate current findings into validated dietary strategies. Overall, while melanoidins represent a compelling target for future research into gut health, their application in functional foods and clinical nutrition awaits more robust evidence from human studies.
